# Does the ultrasound imaging predict lower limb tendinopathy in athletes: a systematic review

**DOI:** 10.1186/s12880-023-01181-5

**Published:** 2023-12-21

**Authors:** Faiza Sharif, Ashfaq Ahmad, Aliha Shabbir

**Affiliations:** 1 University Institute of Physical Therapy, The University of Lahore, Lahore, Pakistan; 2 Faculty of Allied Health Sciences, The University of Lahore, Lahore, Pakistan; 3 Agha Khan University, Karachi, Pakistan

**Keywords:** Achilles’ tendon, Diagnosis, Patellar tendon, Tendinopathy, Ultrasound imaging

## Abstract

**Background:**

To conduct a systematic review looking into the possibility of US imaging to anticipate and identify future patellar or Achilles tendinopathy symptoms.

**Methods:**

The studies that were taken into consideration for this review were prospective studies that employed baseline US imaging of the patellar OR Achilles tendons in asymptomatic patients and follow-up measures of pain and/or function. Two impartial reviewers evaluated the study’s quality using the Critical Appraisal Skills Programme instrument.

**Results:**

Participants in the included studies in this review came from various sports. The systematic review revealed a link between baseline tendon abnormalities in the US and a higher chance of developing both patellar and Achilles tendinopathy as well as their future occurrence. Nine of the included studies examined the patellar tendon alone, eight the patellar and Achilles tendon together, and four the Achilles tendon exclusively. For both tendons, US administration is done in a largely consistent manner. The tendon abnormalities of tendon thickness, hypoechogenicity and vascularity at baseline were associated with an increased risk of both Achilles and patellar tendinopathy.

**Conclusions:**

This systematic review shows that abnormal tendon structures seen by US in asymptomatic persons can predict the development of tendinopathy.

## Introduction

Lower limb tendinopathy is distinguished by activity-related pain that prevents people from engaging in sports and recreational activities [1]. Both MRI and ultrasound can offer detailed morphological information about patients with Achilles and patellar tendons overuse problems. Despite these apparent benefits, structural abnormalities identified by imaging may not exactly match with symptoms [2]. In clinical practice, imaging has typically served as a diagnostic and monitoring tool. Due to its quick, easy, and practical application in sports and other disorders, the use of the US has expanded among musculoskeletal practitioners. Clinically, the US has been used to image the tendons to differentially diagnose, track the effectiveness of therapies, and eliminate the possibility of developing new symptoms in the future. The US has been used to assess painful tendons in athletes and identify structural anomalies such as tendon thickness with hypoechoic regions and increased vascularity [3]. Past prospective investigations have revealed that these structural irregularities raised the likelihood of tendinopathy symptoms developing in the future. Accordingly, it has been proposed that if these abnormalities are identified at baseline, high-risk asymptomatic athletes can be ruled out, and their training regimens and/or interventions can be changed to stop the development of further symptoms [4]. According to many cross-sectional studies assessing tendon structure, the phenomenon of future symptoms developing with structural abnormalities at baseline on imaging is still unclear, and it may be caused by a simple normal physiological response to the increased demands of sports and does not necessarily warrant future symptoms [5]. As a result, when medical experts discover structural abnormalities on imaging, they are unable to decide whether to change the training regimens of athletes or to take other preventative measures. Additionally, there is no standardized scanning process in place. Therefore, the purpose of this systematic review is to examine how ultrasound imaging might be used to anticipate future lower limb tendinopathy symptoms.

## Search strategy and study selection

The study followed the strategy proposed by the Preferred Reporting Items for Systematic Reviews and Meta-Analysis (PRISMA) statement [6]. The institutional review board granted ethical approval (IRB-UOL-FAHS/829–1/2021) for data confidentiality. In January 2021, databases were used to build and conduct a thorough search strategy: Cumulative Index to Nursing and Allied Health Literature (CINHAL), MEDLINE, SPORTDiscs, AMED, EMBASE. The four categories of keywords were merged in search strategy: (1) US (2) tendinopathy (3) Achilles and patellar and (4) cohort/ prospective studies. The search strategies used MeSH terms and free terms combined with Boolean operators AND, OR, NOT. The summary of keywords for the search strategy is given in Table 1.

**Table 1:** Keywords used for search strategy

Tendon/tendon AND	
Knee OR patella AND	
Jumper’s knee OR Patellar tendinopathy OR Tendinitis OR Tendinosis OR Tendinopath OR tendinopathy AND	
Achilles OR Heel OR Tendo calcane OR Tendocalcane OR Tendoachilles OR Tendo achilles OR Achilles tendinitis AND	
Ultrasound OR Ultrasonograph OR Sonograph OR UTC OR Ultrasonic imaging OR Diagnostic Ultrasound imaging AND	
Risk OR Predict OR Associate OR Relate OR Correlate OR Develop OR Prognosis OR Prospect OR Longit OR OR Future OR Characters OR Grade OR Grading OR Classification OR Classify OR Staging	

### Inclusion criteria

The following were characteristics of prospective studies in which US imaging was utilized to predict patellar or Achilles tendon structure seen at baseline [7].

- ▸ To determine the likelihood of developing patellar or Achilles tendinopathy in the future, US readings associated with a clinical outcome measure (pain & functional impairment) are used.
- ▸ The follow-up period had to be at least 24 hours long.
- ▸ The analysis of the tendon structure could have been qualitative or quantitative.
- ▸ Studies must have been published within the last 20 years, in English.
- ▸ Participants in studies could be of any age.
- ▸ Participants with related comorbidities and those with insertional and mid-portion tendinopathy may be included in studies.

### Exclusion criteria

Studies with the following characteristics excluded.

- ▸ Studies focusing solely on the evolution of tissue structural changes without accompanying clinical measurements (as indicated above).
- ▸ Studies that investigated the tendons other the patellar or Achilles tendon.
- ▸ examining the structure of animal tendons.

### Assessment of methodological quality

The Critical Appraisal Skills Programme (CASP) checklist for cohort studies was utilized due to absence of an ideal methodological quality grading tool for prospective research of this kind [8]. There are 12 questions on this checklist; the first two are screening questions, and the following 10 probe the study’s findings, their reliability, and their application to the local population.

For the purposes of this review, questions 2, 7, 8, and 9 were combined because they address related topics. Consequently, seven guiding questions were used to evaluate the included studies. Consistency is crucial when analyzing research because the CASP has numerous factors to consider for each question. As a result, the authors developed and agreed upon a list of criteria for each issue to be taken into account when evaluating the quality of the included studies. Using the listed criteria, two authors independently rated the studies, with a third reviewer mediating any scoring discrepancies. No overall quality score was given to the included studies because the CASP checklist was initially intended to be used as a teaching tool in a workshop environment. Instead, based on these precise criteria, the advantages and disadvantages of each study were evaluated.

### Data extraction

Two reviewers took data from the studies that were included, including information on patients’ demographics, population samples, measures of tendon structure, the number of tendons that developed symptoms among those who had baseline imaging that was normal or abnormal, and the definition of tendon abnormality given in each study. Any variation in tendon structure, such as hypoechogenicity, increased thickness, or increased vascularity as observed on power Doppler US, were all considered to be tendon abnormalities. Data were analyzed collectively due to similarities in the outcome measures utilized, the tendons involved, the participants, and the ability to predict future symptoms. The corresponding authors of the original study were contacted in cases where data weren’t accessible or where the methodology needed to be clarified. The results were not included in the systematic review and were instead presented descriptively if information on tendon structure measurements and the number of tendons among individuals with normal or abnormal imaging at baseline were not readily available.

## Results

### Studies identification

**Fig. 1:**
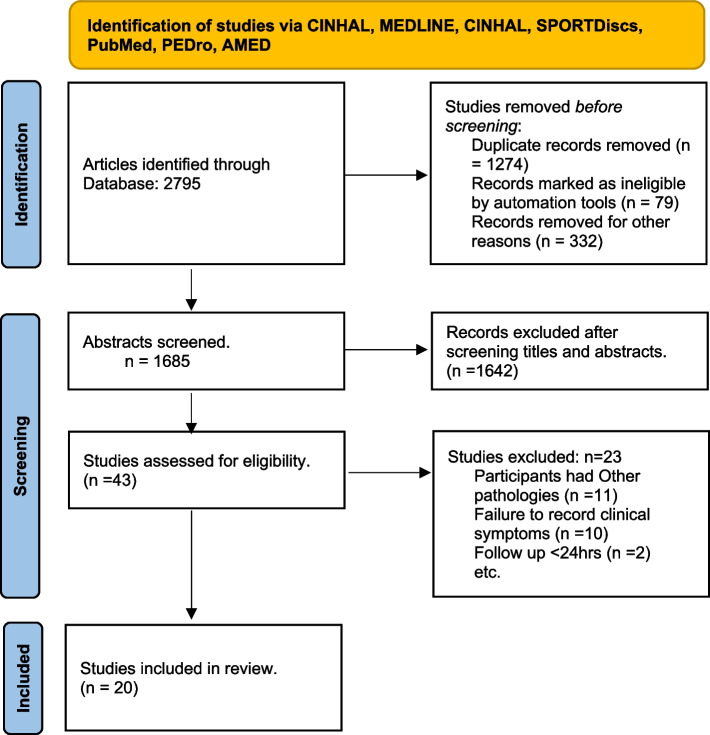
Preferred reporting item for systematic reviews and Meta-analyses (PRISMA) Flow chart

An electronic search yielded 2795 potentially pertinent studies in total. After data from 1274 studies overlapped, they were all excluded. After the screening of titles and abstracts of each study, 43 full-text studies were recognized as potentially relevant studies. Twenty-three further studies were excluded after screening the full text of short-listed studies. The procedure for studies identification is given in Fig. 1.

### Features of the included studies

Table 2 provides a thorough illustration of the selected studies. Participants’ ages ranged from 15 to 66 years, with a similar mean age across all the included studies. Regarding participant characteristics, all but one of the 20 studies that were considered were conducted among athletes. Particularly five involved volleyball, [9, 36–38, 40] three had basketball, [14, 35, 39] three had soccer players, [11, 15, 16] one had elite fencers, [13] three had runners’ population, [12, 17, 18] one involved badminton, [19] and ballet dancers each, [20] and one had different sports playing populations [14]. Patients from the general population participated in one of the included studies [21]. There were exclusively male participants in seven out of the 20 studies, [11, 15–18, 38, 39] and the remaining 13 had participants of both genders. The follow-up time ranged widely, from 2 days to 4 years. An ultrasound was used in every study that was included to look into the tendon structure while only one study included used Ultrasound Tissue Characterization for tendon structure evaluation [36]. Nine studies explored tendon thickness, Hypoechogenicity, and vascularity, [9, 12, 13, 15, 21, 22, 37, 38, 40] six studies investigated tendon thickness and hypoechogenicity, [11, 14, 16, 18, 35, 39] two investigated Thickness, Hypoechogenicity, Intratendinous delamination, and Calcifications, [17, 23] two investigated hypoechogenicity, [20, 36] and one investigated vascularity only [19]. There were many different clinical outcome measures for pain and/or function, including subjective pain, functional capacity (such as VISA- scale), and performance-specific tests like the single-leg squat and plyometric exercises, as well as pain and tenderness on palpation.

**Table 2:** Characteristics of the included studies

Study design	Participant’s demographics	Population	Tendon	Parameter examined	Structural change under the US	US imaging and follow up	References
Prospective cohort study	*n* = 73 M 26.8 ± 4.8 years (Range N/A)	Elite basketball players	Patella	Thickness, Hypoechogenicity	Abnormal:1) hypoechoic areas 2) increased thickness.	US: Initial and follow-up	[9]
Prospective cohort study	*n* = 104 M (age:< 18 years)	Distance runners	Patellar and Achilles	Thickness Hypoechogenicity Vascularity Tendon clefts Intratendinous calcifications	Abnormal: the presence of 1) hypoechogenicity,2) intratendinous delamination (3) paratenon blurring [10],4) calcification, and 5) tendon thickening	US: Initial and follow-up (1,3,6& 12 mon.)	[11]
Prospective cohort study	*n* = 61(23 M/34F) (range 11–18)	elite ballet dancers	Patellar	Hypoechogenicity	Abnormal: hypoechoic area on greyscale ultrasound for 2 or more time points	US: Initial and 6-monthly Follow-up: 2 y	[12]
Cohort study	*n* = 41 (30 M/11F) Mean age 17.2(range 16–18)	Elite junior volleyball players	Patellar	Hypoechogenicity	Abnormal: Presence of Hypoechogenicity (undefined).	US: Initial and follow-up	[7]
Cohort study	*n* = 158 (84 M/74F) Mean age 17(Range N/A)	Elite junior volleyball players	Patellar	Thickness Hypoechogenicity Vascularity	Abnormal: Presence of (1) Hypoechogenicity (undefined), or (2) increased vascularity (≥stage 2) as defined by Gisslen et al. (2007)	US: Initial and 6-monthly Follow-up: 4 y (average: 1.7 y)	[8]
Cohort study	*n* = 41 (25 M/16F) Mean age 37.25	Marathon runners	Achilles	Thickness Hypoechogenicity Vascularity	Abnormal: Presence of (1) increased tendon thickness (undefined), or (2) Hypoechogenicity (≥grade 2) according to a defined three-point scale (grade 1–3), or (3) vascularity (≥grade 2) according to a defined three-point scale (grade 1–3)	US: Initial (pre-race 1 wk) and 3 d post-race Follow-up: 10 d	[13]
Cohort study	*n* = 37 (15 M/22F) Mean age 27.2 [11–31]	Elite fencers	Achilles and patellar	Thickness Hypoechogenicity Vascularity	Abnormal: Presence of (1) increased thickness (undefined), or (2) Hypoechogenicity (undefined), or (3) increased vascularity (≥stage 2) as defined by Gisslen et al	US: Initial and follow-up: Average 3y	[16]
Cohort study	*n* = 79 (35 M/44F) Mean age 27.4 [10–13, 16–34]	Professional Ballet dancers	Achilles and patellar	Thickness Hypoechogenicity Vascularity Tendon clefts Intratendinous calcifications	Abnormal: Presence of (1) Hypoechogenicity, or (2) increased thickness, or (3) vascularity, or (4) intratendinous calcifications (all undefined)	US: Initial visit Follow-up: 24 mo.	[20]
cohort study	*N* = 86 (56 M, 30 F) Mean age: 21.7 (range N/A)	Badminton	Achilles patellar Quadriceps.	Vascularity	Abnormal: Presence of increased vascularity (≥grade 1) according to a defined six-point scale (grade 0–5).	US: Initial and follow-up: 8 mo.	[17]
Cohort study	*n* = 634 (425 M/209F) Mean age 41.2(17–73)	Long-distance runners	Achilles	Thickness Hypoechogenicity Vascularity	Abnormal: Presence of (1) increased thickness (undefined), (2) Hypoechogenicity (undefined), or (3) presence of vascularity according to a defined five-point scale.	US: Initial visit Follow-up: 12 mo.	[19]
cohort study	*n* = 18 M (Mean age 23.5(22–27.5)	Elite soccer players	Achilles	Thickness Hypoechogenicity Vascularity	Abnormal: Presence of (1) increased thickness > 1 mm, or (2) Hypoechogenicity > 1 mm, or (3) paratenon blurring, or (4) vascularity (undefined).	US: Initial visit Follow-up: 12 mo.	[35]
Cohort study	*n* = 58 (36 M/22F) (range N/A)	Elite and recreational volleyball players	patellar	Thickness Hypoechogenicity Vascularity	Abnormal: Presence of (1) increased thickness (undefined), (2) Hypoechogenicity (undefined), or (3) vascularity of at least one vessel in the sagittal plane > 1 mm in length.	US: Initial and monthly Follow-up: 5 mo.	[36]
RCT	*n* = 207 M (Mean age 25)	Professional soccer players	Achilles and patellar	Thickness Hypoechogenicity	Abnormal: Presence of (1) thickness > 0.5 mm in the Achilles and patellar tendon, or (2) Hypoechogenicity > 0.5 mm in the Achilles tendon and > 1 mm in the patellar tendon.	US: Initial and follow-up: 12 mo	[15]
Cohort study	*N* = 22 (11 M, 11 F) Mean age: 16.3 (15–16 at start)	Elite junior volleyball players	Patellar	Thickness Hypoechogenicity Vascularity	Abnormal: Presence of (1) increased thickness (undefined), or (2) Hypoechogenicity (undefined), or (3) vascularity (≥stage 2) to a defined four-point scale (grade 0–3)	US: Initial, regular intervals and follow-up (6 total) Follow-up: 3 y	[37]
cohort study	*N* = 60 (29 M, 31 F) Mean age: 17.2 [11, 14–16, 35]	Junior volleyball players	Patellar	Thickness Hypoechogenicity Vascularity	Abnormal: Presence of (1) increased thickness (undefined), or (2) Hypoechogenicity (undefined), or (3) vascularity (≥stage 2) according to a defined four-point scale (grade 0–3)	US: Initial and follow-up: 7 mo	[38]
Cohort study	*n* = 45 (27 M/18F) Mean age 42	Patients from a university sports medicine center	Achilles	Thickness Hypoechogenicity Vascularity	Abnormal: Presence of (1) increased thickness > 6 mm, or (2) Hypoechogenicity (undefined) Presence of the above features were graded according to a defined three-point scale.	US: Initial & 12 mo. Follow-up: 24 mo.	[18]
Cohort study	*n* = 54 M (Age range 18–35)	Professional soccer players	Achilles and patellar	Thickness Hypoechogenicity	Abnormal: Presence of (1) thickening > 1 mm, or (2) Hypoechogenicity > 1 mm.	US: Initial and follow-up: 12 mo	[14]
Cohort study	*n* = 24 M (Mean age 27.5 [10–13, 16–34]	Athletes from various sports: basketball, cricket, netball, and Australian rules football	Patellar	Thickness Hypoechogenicity	Abnormal: Presence of (1) thickness, or (2) Hypoechogenicity (all undefined)	US: Initial and follow-up: 47.1 mo. (32 = 80 mo.	[39]
Cohort study	*n* = 26 (8 M/18F) Age range 14–18	Elite Junior basketball	Patellar	Thickness Hypoechogenicity	Abnormal: Presence of (1) thickness, or (2) Hypoechogenicity (all undefined).	US: Initial & follow-up Follow-up: 16 mo (12–24 mo	[40]
Prospective cohort study	*n* = 138 Males Mean age (36.2 ± 12.0 years	Recreational half-marathon and full-marathon runners.	Achilles and patellar	Thickness Hypoechogenicity	Abnormal: Presence of (1) thickness, or (2) Hypoechogenicity.	US: Initial and follow-up: 12 mo	[17]

### Study quality & scoring

Table 3 displays the critical evaluation of included studies using the CASP checklist. Overall, most studies met the inclusion criteria. Each of the included studies employed a representative sample size and recruited people in accordance with predetermined suitable inclusion criteria. The control of confounding factors, such as the use of blinding and/or regulation of training load, was one of the key limitations of the studies that were included. There were differences in the methodological quality about the proper follow-up of the included participants. The above-mentioned wide heterogeneity in how pain and/or function were assessed is a last methodological point of concern. The completeness, interpretation, and generalizability of the results may have all been impacted because several studies did not fit the criteria. However, in general, each of the included research used adequate study designs, sound methodological quality, and had stated objectives.

**Table 3:** Critical appraisal summary of the included studies using CASP checklist for cohort studies

Cohort studies													
1	2	3	4	5a	5b	6a	6b	7	10	11	12	Score	References
Y*	Y	Y	Y	Y	Y	Y	Y	Y	Y	Y	Y	100%	[9]
Y	Y	Y	Y	Y	Y	Y	Y	Y	Y	Y	Y	100%	[11]
Y	Y	Y	Y	Y	Y	Y	Y	Y	Y	Y	Y	100%	[12]
Y	Y	Y	Y	Y	Y	Y	Y	Y	Y	Y	Y	100%	[7]
Y	Y	Y	Y	Y	Y	Y	Y	Y	Y	Y	Y	100%	[8]
Y	Y	Y	Y	Y	Y	Y	Y	Y	Y	Y	Y	100%	[13]
Y	Y	Y	Y	Y	Y	Y	Y	N	Y	Y	Y	92%	[16]
Y	Y	N*	Y	N	N	Y	Y	Y	Y	Y	Y	75%	[20]
Y	Y	Y	Y	Y	Y	Y	Y	Y	Y	Y	Y	100%	[17]
Y	Y	Y	Y	Y	Y	Y	Y	Y	Y	Y	Y	100%	[19]
Y	Y	N	Y	N	N	Y	Y	Y	Y	Y	Y	75%	[35]
Y	Y	N	Y	N	N	Y	Y	Y	Y	Y	Y	75%	[36]
Y	Y	Y	Y	Y	N	Y	Y	Y	Y	Y	Y	92%	[37]
Y	Y	Y	Y	Y	N	Y	N	Y	Y	Y	Y	83%	[38]
Y	Y	N	Y	N	N	Y	Y	Y	Y	Y	Y	75%	[18]
Y	Y	Y	Y	N	N	Y	Y	Y	Y	Y	Y	83%	[14]
Y	Y	Y	Y	N	N	Y	Y	Y	Y	Y	Y	83%	[17]
Randomized controlled trial													
1	2	3	4	5	6			7	9	10	11	Score	
Y	Y	N	N	N	N			Y	Y	Y	Y	60%	[15]

*Y = YES *N = NO

Ultrasound protocol administration and results of the included studies are shown in Table 4. Mostly, studies included 1–2 positions to execute ultrasound scans of patellar and Achilles’ tendons: the supine positions and prone positions, respectively. Of the supine position, various knee flexion angles were used: 20degrees, [9, 18, 37] 30degrees, [13, 39] 90degrees, [16, 20] 100degrees, [36] 120degrees, [17] one study used patient in supine with knee extension, [40] while for Achilles the ankle flexion used: 90 degrees with feet hanging over the table [13, 15–17, 21, 22]. One study evaluated anterior knee tendons with supine and 15 degrees knee flexion, [19] five of the included studies did not describe a patient position for an ultrasound scan [13, 16, 20, 23, 36, 37]. Six out of all included studies used the proximal to distal approach for ultrasound scan. While the rest of the studies did not describe the approach used. Only two studies used unilateral ultrasound limb scans, [20, 36] while the rest had a bilateral scan of either patellar or Achilles’ tendons. Most of the studies that were reviewed looked on whether the US could forecast when patellar or Achilles tendinopathy will arise [7]. However, several authors claimed there is either no or little correlation between baseline structural abnormalities and subsequent tendinopathy. One of the studies revealed that there isn’t much of a change in tendon structure (echo types I–IV) during a sporting event, [36] and other studies concluded that ultrasound appearance of the tendon should not be solely responsible for the management of patellar tendinopathy [14]. Furthermore, a study that looked at tendons concluded that there is no connection between structural irregularity and potential tendinopathy [15].

**Table 4:** Ultrasound protocol administration and results of included studies

Patient position	The direction of the scan	Side	Clinical application	Region of interest	Results	Practical applications	References
Patellar: supine position with approximately 30° knee flexion, with a pillow under the popliteal space.	Not described	Bilateral	Monitoring	5 mm distal to the inferior pole of the patella.	Of the 146 tendons, 91 had some degree of sonographic abnormality. Three main patterns were identified: I, II, III.	Patterns of sonographic abnormalities, including NV, demonstrated greater pain. A combination of 2 or more ultrasound abnormalities can determine variations in pain variations among basketball players.	[9]
Achilles: Prone with the feet hanging over the table edge and the ankles flexed to 90°; Patellar: supine with 120° knee flexation	Not described	Bilateral	Predicting	Short-axis images were saved at the tendon location at its greatest width while the longitudinal assessment was made in the midline tendon, centered over the area of maximum thickness.	24.1% of the Achilles tendon had structural abnormalities; and 23.1% of the patellar tendons before the race. The participants with tendon structural were 2–3 times more prone to develop pain within 1 year than those without	25% of the asymptomatic runners had structural changes, which lead to an increased risk of Achilles and patellar tendon pain within 12 months.	[11]
Patellar: patient in supine with 90°knee flexion.	From proximal to distal	Unilateral	Monitoring	1 cm distal to the disappearance of the patellar inferior pole.	During the study, 9% of participants developed tendon pathology, out of which only 2–5% reported tendon pain.	Abnormality in the proximal part of the patella can occur during adolescence	[12]
Patellar: supine, with approx. 100° of knee flexion	Proximal to distal	Unilateral	Monitoring	20 mm distal from the apex of the patella	No remarkable changes in tendon structure (echo types I-IV) over the sports event.	Either the tendon structure is stable enough, UTC is not significant, or decreased tournament/time for considerable change.	[7]
Patellar and quadriceps: supine, with slight knee flexion (20°)	Proximal to distal	Bilateral	Monitoring	The proximal, mid, and distal parts of the tendons	Out of 141 asymptomatic athletes, only 22 athletes (35 patellar tendons) advanced to the jumper’s knee.	The risk factors to develop jumper’s knee among adolescent volleyball athletes were hypoechoic areas and neovascularization at baseline. 7–11% increased quadriceps tendon thickness in healthy athletes, and no change in patellar tendon thickness.	[8]
Achilles: Prone, legs hanging over the edge of the table.	Not described	Bilateral	Predicting	The mid-portion of the free Achilles tendon (2–6 cm proximal to the calcaneal insertion)	A remarkable reduction in tendon stiffness was due to Marathon running (*p* = 0.049) and an increase in Doppler signals (*p* = 0.036). Achilles tendon pain was observed in four out of 21 (19%) runners post-race [VAS 4.0 (±1.9), VISA 74.2 (±10.1)]. Decreased stiffness of the tendon at baseline was correlated with post-marathon Achilles tendon pain (*p* = 0.016).	The prior soft Achilles’ tendon properties seen on; sonoelastography may be a risk factor for the occurrence of symptoms after running.	[13]
Patellar and quadricep; supine with 30° knee flexion; Achilles: the patient prone, the heels overhanging couch, and the ankles flexed to 90°	Proximal to distal	Bilateral	Predicting	10 mm proximal to the superior-posterior aspect of the calcaneus, the patellar tendon 5 mm distal to the patellar attachment, and the quadriceps tendon 10 mm proximal to its patellar insertion	At baseline readings, the abnormal patellar tendon was probably more prone to develop symptoms than those normal (*P* < 0.05, Fisher’s exact test), while US and PD abnormalities on Achilles and quadriceps tendons were not associated with the development of symptoms over a longer duration. A small percentage of tendons diagnosed as normal at baseline (1.45%) exhibited US abnormalities at follow-up of 3 years.	It is questionable that secondary investigations through PD give more information or alter prognosis in patients with a US diagnosis of tendinopathy.	[16]
Not described	Proximal to distal	Bilateral	Predicting	1 cm from both origin and insertion	There was a weak association of moderate or severe hypoechoic defects with future development of symptoms of tendinopathy (*p* = 0.0381); and no correlation between any of the other ultrasound abnormalities and the development of the symptoms.	Ballet dancers have common sonographic abnormalities, but only the presence of focal hypoechoic changes are predictive of future symptoms development in tendons.	[20]
Achilles: Prone position with a pillow under the distal tibia with feet hanging over the table in slight plantarflexion. Anterior knee tendons: supine position with 15° knee flexion with a pillow (relaxed position).	Not described	Bilateral	Predicting	2 cm in the longitudinal direction of the tendon	36% experienced pain in 51 tendons (15%), (*P* = .0002). The abnormal flow was observed in (83%) at the beginning of the season compared with (48%) at the follow-up. (*P* < .0001). (68%). had abnormal flow. (85%) with the abnormal flow at the start of the season were pain-free. At the end of the season, (35%) had abnormal flow. The majority of the tendons (73%) were pain-free and abnormal flow at the beginning of the season was normalized (no pain and normal flow) at the end of the season.	It was impossible to establish any association between intratendinous flow and pain at the beginning of the season or the follow-up (end of the season). Intratendinous flow at the beginning of the season could not predict the symptomatic outcome at the end of the season.	[17]
Achilles: Prone position with the legs of the subjects hanging over the edge of the table and ankles passively flexed at 90°.	Not described	Bilateral	Predicting	Point 3 cm proximal to the calcaneal insertion and at its thickest.	The highest odds ratio (OR) for the appearance of MPT within 1 yr was found for intratendinous blood flow (“neovascularization,” OR = 6.9, *P* < 0.001). The subjects having positive Achilles tendinopathy history were found to have high risk. (OR = 3.8, *P* < 0.001). Another significant parameter was a spindle-shaped thickening of the tendon observed on PDU (Wald χ2 = 3.42).	Healthy runners with the diagnosis of intratendinous microvessels in the Achilles’ tendon on PDU can predict the appearance of MPT symptoms.	[19]
Achilles: Prone with their ankles in a relaxed position (approximately plantar grade).	Not described	Bilateral	Predicting	insertion on the calcaneus (defined on the US as the clearest image of the pre-Achilles bursa); the musculotendinous junction [the area examined on the US where the last soleus fibers attach to the tendon and the midpoint of the two	mid-tendon thickness at baseline was greater (*p* = 0.041) in tendons that had symptoms [median (IQR): 0.53 (0.51–0.55) cm] in the upcoming year than tendons remaining asymptomatic [0.48 (0.45–0.52) cm]	There was no association between the presence of baseline ultrasound signs and future development of symptoms in the upcoming years (Chi-Square: 1.180, *p* = 0.277). A thicker tendon thickness of the mid-portion was considered as a risk factor for future development of Achilles tendinopathy in elite soccer players.	[35]
Not described	Not described	Bilateral	Predicting	Three categories on greyscale imaging; normal, diffuse thickening, hypoechoic	Painful tendons with hypoechoic regions (59%) and contain Doppler flow (42%) than tendons with diffuse thickening (pain in 43% and Doppler flow in 6%)	The transitions identified between normal, diffusely thickened tendons and those containing a hypoechoic region indicate that these greyscale US changes may show different phases of tendon pathology.	[36]
Not described	Not described	Bilateral	Predicting	6 mm from the insertion at the lower patellar pole. The normal Achilles tendons thickness was measured 20 mm from the distal attachment at the calcaneus, and Achilles tendons with increased thickness were measured at the thickest point.	The presence of ultrasonographic tendon abnormalities before the season greatly increased the risk of developing tendon symptoms during the season (relative risk = 1.9; 95% CI, 1.2–3.1; *P* = .009).	With the use of ultrasonography, tendon changes in soccer players can be diagnosed before symptomatic appearance.	[15]
Patellar: Supine, first with the extended knee and then with the slightly flexed knee (20°)	Not described	Bilateral	Predicting	Not described	Development of patellar tendinopathy in 2 of 25 (2 were excluded) tendons that were normal (clinical and US+PD) at inclusion and were also present in six tendons.	Normal clinical tests and ultrasound findings at the start indicated a low risk for these elite junior volleyball players to sustain jumper’s knee during three school years with intensive training and playing.	[37]
Patellar: patient supine with an extended knee.	Not described	Bilateral	Monitoring	Not described	The 20 clinically normal tendons with the normal US and PD sonography at inclusion lead to the structural tendon changes, whereas neovascularisation was developed in 12 tendons.	The clinical diagnosis of patellar tendinopathy is most often accompanied by neovascularisation in the area with structural tendon changes. The finding of neovessels might represent the worsening of the condition.	[38]
Achilles: Prone, and feet hanging over the table in a relaxed position	Not described	Bilateral	Predicting	A transverse scan was used to measure tendon thickness by maximum anteroposterior diameter at a neutral position of the talocrural joint. The tendon was considered a thickened tendon with a diameter greater than 6 mm.	65% of the symptomatic tendons had abnormal morphology on. The US and 68% of asymptomatic tendons had normal morphology. Baseline US findings did not anticipate the 1-year clinical outcome. No improvement in diagnostic qualities of US after the addition of color and power Doppler	In chronic Achilles tendinopathy, moderate correlation with clinical assessment on US and MRI. Association between Graded MRI appearance and clinical outcome, but no association with the US.	[18]
Patellar and Achilles: The ankle and knee flexed 90°.	Proximal to distal	Bilateral	Predicting	Tendons were considered abnormal, 2 to 5 cm proximal from the calcaneal insertion and of more than 1 mm to the normal distal part of the tendon.	During the preliminary examination, 11% of the Achilles tendon had abnormal findings in the US. it was observed that they had a 45% risk of developing symptoms of Achilles tendinosis. At the end of the season, only one of the players with normal tendons developed symptoms of tendinopathy.	For the first time, it is now credible to recognize risk factors for the development of serious tendon disorders in asymptomatic athletes.	[14]
Not described	Not described	Bilateral	Predicting	Not described	Development of hypoechoic area in seven normal patellar tendons at baseline with only two produced symptoms, there is no association between baseline ultrasound changes and symptoms at follow-up.	Management of patellar tendinopathy should not only rely upon ultrasonographic changes; assessment of the clinical features remains the foundation of significant management.	[39]
Not described	Not described	Bilateral	Predicting	Not described	During the study period, ultrasonographic changes were more likely to appear in males than females (*P* < 0.025), with more training hours per week (*P* < 0.01), while half (50%) of abnormal tendons in females became normal as observed on the US.	It was impossible to anticipate the future development or resolution of tendon symptoms by qualitative or quantitative analysis of baseline ultrasonographic images.	[40]
Patellar and quadriceps: supine, with slight knee flexion (20°)	Not described	Bilateral	Monitoring	The proximal, mid, and distal parts of the tendons	Ultrasound abnormalities were significantly associated with approximately a 3-fold increase [hazard ratio (HR) = 2.55, *P* = 0.004] in the hazard of developing pain in the Achilles tendon and patellar tendon (HR = 1.67, *P* = 0.042) over the year after the race.	The presence of ultrasonographic abnormalities is associated with increased development of pain in the Achilles and patellar tendons within 1 year of a marathon or half marathon.	[17]

## Discussion

### Main findings

The findings of this study showed a recurrent trend, which was consistent throughout the included studies, of an elevated risk of Achilles and patellar tendinopathy in the presence of baseline abnormalities of tendon thickness, Hypoechogenicity, and vascularity in the tendons.

### The burden of tendinopathy

While patellar tendinopathy is prevalent, especially in activities involving jumping, Achilles tendinopathy can affect up to 30% of runners [24]. Despite the relatively high occurrence of lower limb tendinopathy, particularly in populations involved in sports, rehabilitation is still time-consuming and has a mixed history of success [25, 26]. This causes frustration from both the standpoint of the athlete and the health professionals. In addition, protracted and occasionally successful rehabilitation in amateur and professional sports places a greater financial strain on players and athletic organizations.

### The role of ultrasound in predicting tendinopathy

Identification of “at risk” athletes is a top priority to try to prevent the detrimental effects of tendinopathy on sporting participation and quality of life, given the significant impact of Achilles and patellar tendinopathy [27]. The preferred imaging modalities for determining tendon dimensions are MRI and US technologies [28]. Particularly in the field of sports medicine and tendon disorders, US has grown in favor among musculoskeletal practitioners, and recent technological improvements have made US more accessible and cheaper [29, 30]. The findings of this systematic review showed a recurrent pattern suggesting that baseline structural abnormalities of tendon thickness, Hypoechogenicity and vascularity in the tendon are related to the emergence of future tendinopathy. As a result, it’s possible that structural abnormalities in populations who are asymptomatic are indicators of early, pre-symptomatic pathology that will eventually manifest as episodes of pain and/or diminished function [31]. These results’ robustness and consistency may have significant effects on the therapeutic treatment and avoidance of patellar and Achilles tendon disorders. Utilizing US to see tendon anomalies may help identify athletes who may be at risk and enable timely intervention through the use of preventative measures like modifying training loads [32] or adopting the proper tendon loading programs [11]. Future research is necessary because there is currently insufficient evidence to justify such techniques.

Given the generally poor link between structure and pain in tendinopathy, intervention may not be indicated in all instances of structurally aberrant tendons identified using US imaging. Studies have found tendon abnormalities in as many as 59% of asymptomatic populations, with the prevalence of these abnormalities rising with age and involvement in sports [10]. Tendon structural abnormalities have been observed in a significant proportion of asymptomatic individuals [10]. Numerous investigations have shown that structural changes in asymptomatic sports populations across the body, in addition to structural abnormalities in tendons, are common. Papavasiliou et al. [33] used MRI to examine structural changes in the hip in asymptomatic gymnasts, the results showed that up to 63% of the sample group had hip “impingement” symptoms. These findings are confirmed in a variety of body parts and among athletes, with structural abnormalities in the shoulder, knee, hip, and spine occurring in as many as 89% of asymptomatic athletes [34, 41–43].

Evidence also points to the possibility that unneeded imaging interventions may have a negative and damaging impact on patients’ perceptions and habits [44]. For instance, one study of low-risk LBP patients found that those who underwent imaging had worse overall outcomes in terms of pain and general health than those who got no imaging [45]. Unfortunately, there has not yet been any comparable studies on tendon problems, thus caution should be used when drawing direct analogies between LBP and tendon disorders. With the rising acceptance and potential benefits of techniques like UTC or elastography, it may be possible to see the tendon anatomy more clearly. The need to weigh the possible negative effects of having an athlete feel as though their body is vulnerable based on what may be typical physiological reactions to the loading demands arises with enhanced visualization. This may point to the critical importance of a clinician’s communication techniques when conveying imaging results to athletic populations. This illustrates how complicated tendinopathy is and how little we understand it [10].

### Clinical implications

Although structural abnormalities are a strong indicator of future tendinopathy development, it can be challenging to interpret this association in a clinical setting due to the prevalence of abnormalities in asymptomatic people. Therefore, rather than serving as the primary predictor of tendinopathy, structural anomalies may instead need to be taken into account as one of numerous risk factors. There are numerous additional intrinsic and extrinsic risk factors for the onset of tendinopathy. Extrinsic risk factors have been identified for training frequency and volume, [46] larger impacts brought on by faster training, [47] a change in surface density, and shock absorption [48]. Modified foot function, [48] decreased ankle dorsiflexion, [49] sex, [35] diabetes, [50] obesity, [51] muscle weakness, [52] and hereditary factors [53] have all been proposed in relation to intrinsic risk variables. Unfortunately, because most studies on these issues have been cross-sectional, it has been difficult to establish a clear cause-and-effect relationship. Because of this, it is still unknown how these factors might interact to predict the development of tendinopathy, despite advances in our understanding of lower limb tendinopathy. One could argue that routine imaging might be expensive and time-consuming given the high prevalence of structural abnormalities in asymptomatic people. Any modality that enables the identification of athletes at higher risk of injury may, however, outweigh the cost or time-consuming aspect of routine imaging given the financial burden and the potentially career-threatening consequences of tendinopathy in sporty populations [54].

As the limitation of the biomedical structure-pain model become more apparent, there is a growing need to examine sports injuries and injury prevention from a biopsychosocial perspective, which takes into account factors like sociodemographic, psychological, lifestyle, and social factors in addition to local tissue damage [55]. Psychosocial problems that have been linked to injury development in athletic populations include sleep disturbances, [56] fatigue, [57] and anxiety [58]. However, in studies that predict injuries, there has been a reluctance to combine intrinsic psychosocial elements—such as training load, strength, or biomechanics—with other conventional biological risk factors, despite the significance of these factors in the development of injury. Prospective studies combining some of the psychological components mentioned above with traditional risk factors like tendon imaging may offer much-needed clarity and understanding in the complex field of tendinopathy, or possibly even sports injury prevention in general.

### Limitations

Reliability is one of the most brought up objections to US imaging. Due to factors like inexperienced operators, non-standardized imaging techniques, and different transducer positions, [59] US is thought to have a larger chance of error or variance when measuring tendon diameters than MRI. A recent systematic review [60] showed that US displays good to exceptional levels of inter-rater and intra-rater reliability in evaluating tendon thickness and cross-sectional area, despite the fact that operator experience varied greatly among the included investigations.

The inconsistent wording used to describe what is considered a structurally “abnormal” tendon when examined via US is another potential weakness of this review. For instance, Comin et al. [23] employed hypoechoicity (mild, moderate, severe) or its absence to identify abnormalities. Greater than 1 mm of tendon thickening was deemed abnormal in three studies [11, 15, 16]. A tendon needed to be thicker than 3 mm in one study to be classified as abnormal, [22] whereas a tendon needed to be thicker than 6 mm in another study to be classified as abnormal [21]. Because of the wide variation in what constitutes an abnormal tendon among the studies that were included, it is possible to overestimate or underestimate the association between structure and potential future symptoms. Another limitation is to the study populations that were examined; the bulk of them focused on communities of athletes. When extrapolating results to non-athletic populations, care should be used because Achilles and patellar tendinopathy are common in non-athletic populations.

The absence of gold standard diagnostics for detecting tendinopathy is another possible limitation. Regarding the best clinical diagnostic test for tendinopathy, there is a lack of agreement in the research and clinical domains. It is challenging to determine the diagnostic value of US findings because of the broad variance in diagnostic tests utilized in the research in this evaluation and the lack of a commonly recognized gold standard test. Finally, several studies in this review investigated the Doppler US ability to predict tendon vascularity. When employing Doppler settings, temperature is a key confounding variable [61]. However, none of the included Doppler US studies showed that they were adjusting for this confounding factor. However, as only one study used vascularity as the sole indicator, it is doubtful that this concern had an impact on the results.

### Future implications

This study implies that ultrasonography could be used to identify athletes and sportsmen who are more likely to have tendon pathology. In order to reduce the chance of discomfort, these “high-risk” sportsmen could be assessed for biomechanical and training risk factors and given recommendations for training plans or preventative exercise interventions, as well as for quicker rehabilitation should they start to experience symptoms. There is currently no evidence that preventative workouts based on these abnormalities are useful. The logical next step in study is to uncover biomechanical and training risk factors for the development of tendon pathologies in athletes.

## Conclusion

According to the findings of this systematic review, tendon anomalies are a strong indicator of future Achilles or patellar tendinopathies. This could have significant therapeutic ramifications for the treatment and prevention of tendon diseases. The relationship between tendon anomalies and the emergence of subsequent symptoms, however, was only mild. Furthermore, imaging results should only be considered as one element of the clinical prediction of tendinopathy due to the high prevalence of tendon anomalies in asymptomatic tendons.

## Data Availability

All data generated or analyzed during this study are included in this article.
